# Behavioral mechanisms uncovered: the causal effects of service experience design on green consumption via cognitive-affective dual processing

**DOI:** 10.3389/fpsyg.2026.1750281

**Published:** 2026-03-24

**Authors:** Wenhao Li, Kuan Yan

**Affiliations:** 1 College of Economics and Management, Nanjing University of Aeronautics and Astronautics, Nanjing, Jiangsu, China; 2 School of Design and Art, Shaanxi University of Science and Technology, Xi’an, Shaanxi, China

**Keywords:** emotional connection, environmental awareness, green consumption behavior, service experience design, sustainable consumption

## Abstract

**Introduction:**

With the rise of eco-friendly purchasing trends, understanding the mechanisms that drive consumers’ sustainable behaviors has become increasingly important. This study investigates how service design influences sustainable consumption through two psychological pathways: ecological consciousness and affective bonding.

**Methods:**

Using survey data from 582 Chinese urban customers in eco-retail and food service contexts, a multi-component experience framework was developed. Mediation regression analysis was employed to examine how different dimensions of service design affect consumers’ green consumption tendencies.

**Results:**

The empirical results indicate that three service experience dimensions–environmental perception, process facilitation, and interactive responsiveness–significantly enhance consumers’ green consumption tendencies. Furthermore, environmental awareness and emotional connection exert stable partial mediating effects along the influence pathways, jointly forming a dual psychological mechanism characterized by parallel cognitive and affective drivers.

**Discussion:**

These findings demonstrate that experiential service frameworks can shape sustainable consumption decisions by simultaneously activating cognitive recognition and emotional attachment, providing strategic implications for enterprises seeking to promote sustainability-oriented consumer engagement.

## 1 Introduction

Under the impetus of the global sustainable development agenda, green consumption has gradually become a crucial indicator for measuring national environmental governance capacity and citizens’ ecological awareness. With the deepening of ecological civilization construction, consumers have demonstrated significantly increased attention toward eco-friendly products and green services in daily life (Wang et al., 2023). However, the intrinsic driving mechanisms of green consumption behavior remain complex and multifaceted, as traditional explanatory frameworks based on economic incentives or moral norms struggle to fully reveal the psychological foundations behind individual behavioral choices (Diddimani, 2025; Cheng et al., 2024). In green consumption scenarios, service experience—a critical external trigger in consumer decision-making—has not yet been systematically analyzed regarding its impact mechanisms. This study therefore aims to investigate how service experience design can profoundly motivate green consumption behavior by activating consumers’ environmental awareness and emotional connections, thereby elucidating the dual psychological mechanisms underlying green consumption.

Current academic research on green consumption behavior predominantly focuses on individual-level variables such as environmental attitudes, value systems, and social norms, demonstrating that moral cognition and environmental responsibility significantly influence behavioral intentions (Duong, 2023). Concurrently, some studies incorporate service marketing perspectives, emphasizing the role of customer experience and satisfaction in green product adoption (Djunaid, 2024). Building on this foundation, emerging research has begun examining service touchpoints in green consumption contexts, revealing that service process transparency and employees’ green behaviors enhance consumer trust and purchase willingness (Rashid and Lone, 2024; Kwok and Lin, 2024). Nevertheless, these studies rarely systematically explore the motivational function of service experience design in green consumption decisions, lacking quantitative analysis of specific service perception dimensions. Moreover, existing research overlooks potential psychological mediation mechanisms during green consumption, failing to uncover the intrinsic pathways linking service experience with behavioral responses. To address these gaps, this study proposes a dual-mediation model incorporating environmental awareness and emotional connection along the “service experience–psychological mechanism–green behavior” pathway, systematically examining the indirect impact mechanisms of service experience design on green consumption behavior. Utilizing questionnaire surveys, we collected 582 valid samples and conducted empirical analyses through stepwise regression and multiple mediation effect models. The results indicate that service experience design not only directly and positively influences green consumption behavior but also generates significant indirect effects by stimulating consumers’ environmental awareness and emotional connections. Theoretically, this study deepens the understanding of green consumption behavior formation mechanisms. The proposed “service experience design–psychological mechanism–behavioral response” model extends green consumption research from singular cognitive frameworks to multidimensional psychological mechanisms. Practically, the research provides actionable design optimization pathways for green service enterprises, demonstrating effective strategies to motivate green behavior through enhanced environmental consciousness and emotional attachment. These findings offer theoretical support and empirical references for green brand building, service process reengineering, and consumer psychological guidance.

The structure of this paper is organized as follows: section 1 serves as the introduction, clarifying the research background, questions, and objectives; section 2 presents the literature review, synthesizing current research progress on the psychological effects of service experience design and identifying existing research gaps; section 3 details the research design, including data sources, variable specification, and model construction methods; section 4 reports the empirical analysis results; section 5 provides the discussion, examining both theoretical implications and practical insights based on the findings; section 6 concludes the paper by summarizing key discoveries and proposing future research directions.

## 2 Literature review

### 2.1 Theoretical foundations

The formation mechanism of green consumption behavior has long been systematically examined within the domains of behavioral theory and environmental psychology. The Theory of Planned Behavior (TPB) proposed by Ajzen (1991) posits that individual behavior is determined by cognitive structures, including attitudes, subjective norms, and perceived behavioral control. Among these components, attitude serves as a core predictor of behavioral intention. In green consumption contexts, environmental awareness can be conceptualized as an attitudinal cognition reflecting individuals’ perceptions of environmental responsibility and sustainable values, thereby exerting substantial explanatory power over behavioral decision-making. Wang et al. (2024) further extended the TPB framework to the domain of sustainable consumption, empirically confirming the critical roles of environmental attitudes and intrinsic motivation in shaping green purchase intentions. Yeh et al. (2021), in their study on green hotel consumption, validated the applicability of the TPB framework in environmental service settings and demonstrated that cognitive attitudes influence actual behavior through intention formation mechanisms. Accordingly, incorporating environmental awareness as a mediating variable between service experience and green consumption behavior is not only empirically justified but also theoretically grounded in the TPB framework.

At the structural level, Eroglu et al. (2001) developed a behavioral explanation framework for online retail settings based on the Stimulus–Organism–Response (SOR) model, arguing that external environmental stimuli affect individuals’ internal psychological states, which subsequently generate approach or avoidance behaviors. This model provides a structured analytical pathway for understanding how service experiences influence consumer behavior. Han et al. (2022) applied the SOR framework to sustainable consumption scenarios and found that situational stimulus variables indirectly promote green purchasing decisions by shaping psychological perceptions and value identification. This theoretical perspective offers a clear conceptual foundation for defining service experience design as the “stimulus” (S), environmental awareness and emotional connection as “organism states” (O), and green consumption behavior as the “response” (R). Therefore, the overall model structure of this study can be coherently embedded within the SOR theoretical framework.

Beyond cognitive pathways, green consumption decisions are often simultaneously shaped by rational evaluation and affective responses. Within the Dual-Process Theory framework, Evans and Stanovich (2013) argued that individual decision-making typically involves both a reflective processing system and an affective-intuitive system, which operate in coordination across different contexts. Petrescu et al. (2025), in an empirical study on sustainable consumption, further demonstrated that the formation of green consumption behavior relies not only on rational cognitive mechanisms but also significantly on emotional resonance and psychological belonging, thereby explaining discrepancies between intention and actual behavior. Building upon this theoretical foundation, the present study conceptualizes environmental awareness as the rational-cognitive pathway and emotional connection as the affective-processing pathway. Within the dual-process framework, a parallel cognitive–affective dual mediation mechanism is constructed to systematically elucidate how service experience influences green consumption behavior.

### 2.2 Psychological mechanisms of service experience design

As the service economy transitions toward an experience-oriented and emotionally driven paradigm, the theoretical focus of service design has shifted from functional fulfillment to the deep psychological engagement of individuals (Bouman and Simonse, 2023). Experience economy theory suggests that users’ perceived value in services stems not only from outcome-based satisfaction but also from the situational atmosphere, interaction dynamics, and emotional resonance experienced throughout the service journey (Zhu et al., 2022). Consequently, service experience is redefined as a multidimensional psychological process integrating perceptual cognition, emotional response, and social relationship construction, reflecting a high degree of coupling between space, context, and user psychology. Meanwhile, interdisciplinary integration is accelerating a psychological turn in service design methodologies, positioning users not merely as decision-makers but as central agents in the generation of psychological meaning (Kwon et al., 2023; Chen et al., 2023). This shift has spurred extensive research on spatial psychological construction, emotional regulation mechanisms, and user co-creation, gradually forming a composite research framework on the “psychological effects of service experience.”

Recent studies on service spaces as mediums for psychological experience transformation have increasingly emphasized the psychological interaction mechanisms between space and context. Ng and Chen’s (2022) research on cultural heritage settings reveals that through interdisciplinary design approaches (such as integrating humanistic narratives with spatial scenography), spaces can become experiential mediums that enhance users’ emotional connections and cultural belonging. Intriago et al. (2023), through bibliometric analysis, indicate that the psychological linkage between physical environments and service perception has become a global research hotspot and trend in service design. Kille et al. (2023) demonstrate in digital service contexts that through mobile interface and user flow design experiments, service spaces maintain significant psychological guidance capabilities across multidimensional mediums, where users’ transition rhythms between different touchpoints influence their perception of completeness and sense of control. Furthermore, Li et al. (2024) observe that traditional psychological counseling spaces are evolving into “experience venues,” where spaces no longer merely passively contain services but actively become crucial nodes for users’ psychological meaning-making through approaches like “shared goals,” “contextual reconstruction,” and “interaction rhythms.” This transformation expands the psychological functions of service spaces across both structural and symbolic dimensions. Mages and Neely (2023) further introduce the dimension of “temporal experience,” proposing that subjective time perception plays a pivotal role in users’ service evaluation, while traditional service blueprints often lack depictions of “temporal emotions,” limiting the immersive depth of service experiences.

The dimensions of emotional management and social psychology during service processes have also gained increasing attention. Emotions are no longer viewed as byproducts of service delivery but as critical variables influencing customer behavioral motivations. Jang et al. (2022) empirical study in South Korea demonstrates that through “Patient Experience-based Service Design” (PEN-SD) training, clinical nurses showed significant improvement in empathy levels, indicating that emotional perception capabilities can be systematically shaped through service design methods. Chua and Yin (2023), in a Singaporean healthcare management program, found that service design not only enhanced employee satisfaction but also strengthened organizational identification and emotional bonds among teams, with this “design-driven emotional alignment” being a key mechanism for sustaining high-quality service delivery. Murrie and Anderson’s (2023) retail industry research further confirms that optimizing service touchpoints enhances employee well-being and positive emotional states, thereby improving the quality of emotional output in customer interactions. Da Cunha and da Silva (2023) propose the concept of “self-service avoidance psychology,” revealing that users’ resistance to automated service devices (e.g., ATMs) stems from three psychological mechanisms—responsibility perception, environmental pressure, and social evaluation—and emphasizing the need to redesign service contexts based on users’ psychological safety. Junaid et al. (2024) study in Pakistani shopping malls also demonstrates that emotional triggering strategies in service design significantly enhance customers’ “dwell desire” and “revisit intention,” highlighting the direct impact of emotionalized service spaces on customer retention.

In addition to spatial and emotional factors, the psychological dimensions of user participation mechanisms in service design have also garnered significant research attention. Recent studies have begun to transcend the traditional boundaries between “service recipients” and “service providers,” exploring the psychological needs and motivations of users as co-creators of experiences. Raviselvam et al. (2022), in their study on integrating designer values with user needs, proposed that the interplay between psychological needs and personal values in experiential pathways can be made explicit through structured design tools, thereby fostering more empathetic user engagement. Krüger et al. (2023) emphasized the importance of fulfilling employees’ psychological needs (e.g., value recognition, self-expression) in the design process, highlighting the moderating effect of organizational culture on participation motivation. Pratap et al. (2023) focused on post-pandemic e-commerce service environments, noting that consumers’ psychological expectations for “participatory control” have significantly increased, necessitating dynamic feedback and process transparency in service design to enhance users’ sense of control and expectation consistency. Musulin and Strahonja (2023) introduced a unified design framework integrating user experience, business models, and service logic, emphasizing users’ “psychological dominance” in value proposition construction. Hewitt et al. (2025) applied the Experience-Based Co-Design (EBCD) model to co-create psychological intervention services with intellectually disabled individuals, finding that users’ psychological safety and sense of belonging were significantly enhanced during collaborative participation, resulting in service solutions with greater acceptability and emotional resonance.

Existing research has systematically revealed the mechanisms linking service experience design with user psychology across multiple dimensions, including spatial construction, emotional regulation, and co-creation, providing a theoretical foundation for this study. However, while green consumption behaviors inherently possess spatial expressiveness and emotional influence, few studies have systematically analyzed how their experience design leverages psychological mechanisms to incentivize behavior.

**FIGURE 1:**
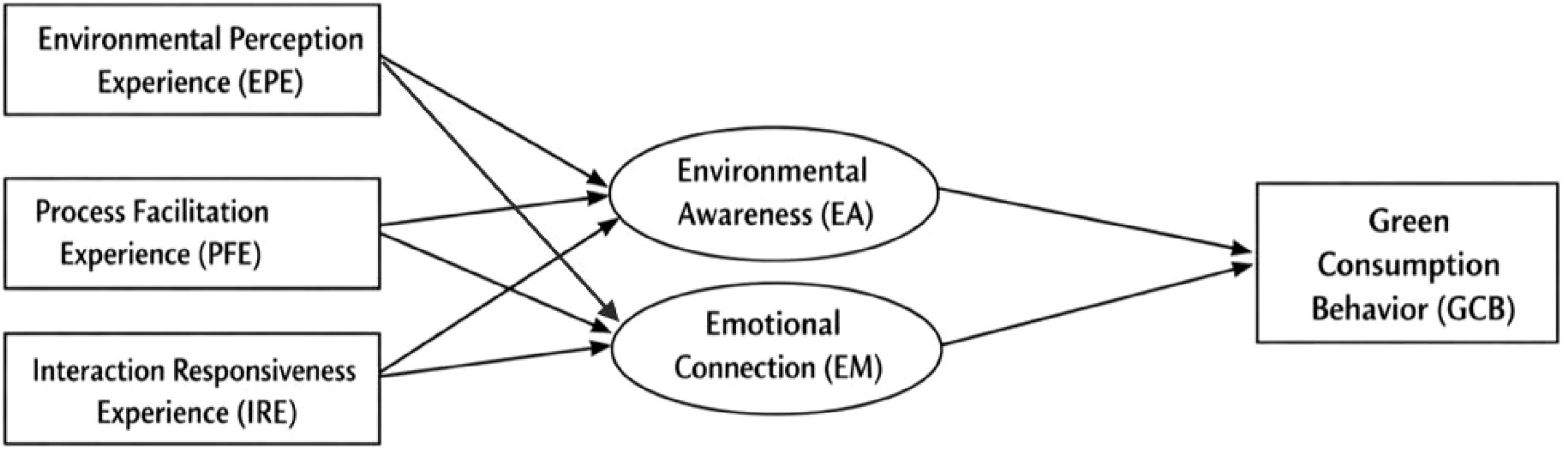
Conceptual framework of the study.

To address this gap, this study adopts a green consumption perspective to construct a service experience model encompassing three dimensions—environmental perception, process convenience, and interactive responsiveness—while introducing environmental awareness and emotional connection as dual mediating variables. The aim is to elucidate how service experiences promote green behavior formation through cognitive-affective synergistic pathways. Based on this theoretical logic, a conceptual research framework is proposed, as illustrated in Figure 1, which visually presents the hypothesized relationships among service experience dimensions, mediating psychological mechanisms, and green consumption behavior.

## 3 Research design

### 3.1 Data sources

The data utilized in this study were collected through a questionnaire survey conducted in green consumption contexts in a Chinese city from July to August 2024. The target participants were consumers engaged in green consumption activities, including but not limited to customers of eco-supermarkets, sustainable fashion stores, and green catering establishments. To ensure sample representativeness and coverage, the questionnaire employed a hybrid online-offline distribution approach. Online dissemination utilized social media platforms and green consumption communities, while offline surveys were administered by trained investigators in physical stores to guide respondents during on-site completion. A total of 320 offline and 308 online questionnaires were distributed. After excluding invalid responses due to abnormal completion times, incompleteness, or logical inconsistencies, 582 valid questionnaires were retained (291 offline; 291 online), yielding a response rate of 92.7%. The survey questionnaire incorporated multiple 5-point Likert scale items focusing on service experience design elements, environmental awareness, emotional connection, and green consumption behavior to measure participants’ cognitive and behavioral responses across these dimensions.

Given that the data in this study were collected through a single-wave questionnaire survey, potential common method bias (CMB) was addressed at both the procedural and statistical levels. Procedurally, the questionnaire was administered anonymously, and respondents were explicitly informed that there were no right or wrong answers, thereby reducing evaluation apprehension and social desirability tendencies. In addition, measurement items for different constructs were randomly arranged to mitigate consistency motifs in responses. Statistically, Harman’s single-factor test was conducted by performing exploratory factor analysis on all measurement items. The results indicate that the first unrotated factor accounts for 32.47% of the total variance, which is below the critical threshold of 40%, suggesting that serious common method bias is unlikely to threaten the validity of the empirical findings.

### 3.2 Variable selection

The core explanatory variable in this study is Service Experience Design (SED), representing the perceived overall experiential quality by consumers in service scenarios. SED is decomposed into three dimensions: Environmental Perception Experience (EPE, weight 0.374), Process Facilitation Experience (PFE, weight 0.341), and Interactive Responsiveness Experience (IRE, weight 0.285), reflecting the ambiance of service spaces, the smoothness of service processes, and the professionalism of staff interactions, respectively. The explained variable, Green Consumption Behavior (GCB), measures environmentally oriented behaviors exhibited by consumers during actual consumption. Its scoring integrates overall levels of agreement regarding green product choices, energy-saving actions, and sustainable consumption attitudes. Mediating variables include Environmental Awareness (EA) and Emotional Connection (EM). EA denotes individuals’ cognition of environmental protection importance and their psychological commitment to environmental responsibilities, while EM reflects affective dependencies, psychological sense of belonging, and loyalty intentions toward brands or service environments. Four control variables—gender (SEX), income level (INC), and green product contact frequency (GPF)—are included to mitigate confounding effects from other factors. All variable names and quantification methods are listed in Table 1.

**TABLE 1:** Variable names and quantification methods.

Variable type	Variable name	Measurement
Independent var.	Service experience design (SED)	Mean score (Likert 5-point scale)
Dependent var.	Green consumption behavior (GCB)	Mean score (Likert 5-point scale)
Mediating var.	Environmental awareness (EA)	Mean score (Likert 5-point scale)
	Emotional connection (EM)	Mean score (Likert 5-point scale)
Control var.	Gender (SEX)	1 = Male, 0 = Female
	Education level (EDU)	1 = College or below, 2 = Bachelor, 3 = Master or above
	Income level (INC)	Monthly income per capita (unit: thousand yuan)
	Green product exposure (GPF)	Mean score (Likert 5-point scale)

### 3.3 Model construction

To systematically analyze the impact pathways of service experience design (SED) on green consumption behavior (GCB) and verify the mediating mechanisms of environmental awareness (EA) and emotional connection (EM), this study employs stepwise regression and a multiple mediation effects model for empirical investigation. The models are specified as follows:

1. Direct effect model

A baseline regression model is established to examine the direct influence of SED on GCB:


$$G\,C\,B_{i}=\alpha_{0}+\alpha_{1}\cdot S\,E\,D_{i}+\overset{n}{\underset{k=1}{\sum }}\beta_{k}\cdot C\,o\,n\,t\,r\,o\,l_{k\,i}+\varepsilon_{i}$$ (1)

Here, *GCB*_*i*_ represents the green consumption behavior score of the i-th sample, *SED*_*i*_ denotes the service experience design score, *Control*_*ki*_ stands for the k-th control variable of the i-th sample, and ε_*i*_ is the random disturbance term.

2. Mediation effect model

To examine whether the dual psychological mechanisms mediate the relationship between service experience design and green consumption behavior, environmental awareness (EA) and emotional connection (EM) are introduced as mediating variables, and the following multiple mediation models are constructed:

Environmental awareness path model:


$$E\,A_{i}=\gamma_{0}+\gamma_{1}\cdot S\,E\,D_{i}+\overset{n}{\underset{k=1}{\sum }}\beta_{k}\cdot C\,o\,n\,t\,r\,o\,l_{k\,i}+\varepsilon_{i}$$ (2)

Emotional connection path model:


$$E\,M_{i}=\theta_{0}+\theta_{1}\cdot S\,E\,D_{i}+\overset{n}{\underset{k=1}{\sum }}\beta_{k}\cdot C\,o\,n\,t\,r\,o\,l_{k\,i}+\varepsilon_{i}$$ (3)

Finally, both mediating variables are incorporated simultaneously to establish a comprehensive regression model for green consumption behavior:


$$G\,C\,B_{i}=\delta_{0}+\delta_{1}\cdot S\,E\,D_{i}+\delta_{2}\cdot E\,A_{i}+\delta_{3}\cdot E\,M_{i}+\overset{n}{\underset{k=1}{\sum }}\beta_{k}\cdot C\,o\,n\,t\,r\,o\,l_{k\,i}+\varepsilon_{i}$$ (4)

Here, *EA*_*i*_ represents environmental awareness, and *EM*_*i*_ denotes emotional connection. If δ*2* and δ*3* are significant, and the coefficient δ*1* decreases or becomes insignificant compared to Model 1, it can be concluded that partial or full mediation effects exist.

## 4 Results and analysis

### 4.1 Measurement model assessment

The core variables in this study are latent psychological constructs measured using Likert-scale items. Prior to conducting structural path analysis, it is necessary to assess the reliability and validity of the measurement model. Regarding reliability (see Table 2), the Cronbach’s α and Composite Reliability values for all latent constructs exceed the recommended threshold of 0.70, indicating satisfactory internal consistency of the measurement scales. In terms of convergent validity, the Average Variance Extracted (AVE) values for all constructs are above the recommended benchmark of 0.50. Moreover, all standardized factor loadings are statistically significant, suggesting that the measurement indicators effectively capture their corresponding latent variables.

**TABLE 2:** Reliability and convergent validity results.

Construct	Cronbach’s alpha	Composite reliability (CR)	AVE	Factor loadings
SED	0.882	0.908	0.624	0.721–0.842
EA	0.854	0.889	0.601	0.703–0.818
EM	0.869	0.903	0.648	0.734–0.861
GCB	0.836	0.878	0.587	0.692–0.823

For discriminant validity assessment (see Table 3), the Fornell–Larcker criterion was applied. The results indicate that the square root of the AVE for each construct exceeds its correlations with other constructs, demonstrating satisfactory discriminant validity among the variables. Overall, the measurement model meets the statistical requirements in terms of reliability and validity, thereby providing a solid and credible foundation for subsequent structural model analysis.

**TABLE 3:** Discriminant validity.

Construct	SED	EA	EM	GCB
SED	0.790	0.775	0.805	0.766
EA	0.452			
EM	0.438	0.511		
GCB	0.487	0.528	0.503	

### 4.2 Descriptive statistics

As shown in Table 4, the average value of green consumption behavior (GCB) is 3.754 with a standard deviation of 0.586, indicating that the overall green behavior level of the sample population falls within the moderate-to-high range, with certain individual variations. This might be related to the enhanced dissemination of green consumption concepts in recent years. The service experience design (SED) has a mean of 3.842 and a standard deviation of 0.619, suggesting that most respondents rated the quality of service experiences they received relatively highly. This could stem from the fact that the sample primarily originates from service scenarios with standardized management and mature experience design. Among the three dimensions of SED, environmental perception experience (EPE) has a mean of 3.902, process-facilitation experience (PFE) of 3.837, and interaction responsiveness experience (IRE) of 3.781—all at moderate-to-high levels. This reflects respondents’ overall high satisfaction with the environmental ambiance, process design, and interaction quality in green service scenarios.

**TABLE 4:** Descriptive statistics.

Variable	Mean	SD	Minimum	Maximum
GCB	3.754	0.586	1	5
SED	3.842	0.619	1	5
EPE	3.902	0.601	1	5
PFE	3.837	0.582	1	5
IRE	3.781	0.614	1	5
EA	3.693	0.552	1	5
EM	3.725	0.575	1	5
SEX	0.416	0.494	0	1
EDU	1.872	0.487	1	3
INC	4.117	1.296	1.5	8
GPF	3.317	0.968	1	5

Environmental awareness (EA) has a mean of 3.693 and a standard deviation of 0.552, demonstrating that the sample population is generally sensitive to environmental knowledge and responsibility recognition. This is likely closely tied to the improvement of societal environmental education. Emotional connection (EM) has a mean of 3.725 and a standard deviation of 0.575, indicating that consumers generally exhibit a degree of emotional attachment during service interactions. This may correlate with the emotional communication and cultural value design embedded in green brands or service scenarios. For the control variables, the demographic characteristics exhibit reasonable distribution, ensuring representativeness and discriminative validity.

### 4.3 Baseline regression results

The baseline regression results of service experience design (SED) on green consumption behavior (GCB) are presented in Table 5. All three core dimensions of SED—environmental perception experience (EPE), process-facilitation experience (PFE), and interaction responsiveness experience (IRE)—exert significantly positive effects on GCB. The regression coefficient for EPE is 0.157, significant at the 1% level, indicating that when consumers distinctly perceive the transmission of green concepts during service interactions—such as the use of eco-friendly materials, energy-efficient and low-carbon operational practices, or visual representations of green culture—they are more likely to develop recognition and inclination toward green consumption. The regression coefficient for PFE is 0.142, also significant at the 1% level. This suggests that service processes characterized by high efficiency and convenience, such as seamless payment procedures, transparent product information, and user-friendly return policies, not only enhance consumption experiences but also provide behavioral facilitation for green decision-making, reducing the time costs and psychological burdens associated with implementing green behaviors. The regression coefficient for IRE is 0.129 (*t* = 4.117), also significant at the 1% level. These results demonstrate that timely responses to consumer demands, personalized communication, and green consultation services during service interactions improve consumers’ subjective evaluations of service quality and establish stronger psychological alignment with green values, thereby further promoting the translation of green intentions into behavior.

**TABLE 5:** Baseline regression analysis results.

Variable	GCB	VIF
EPE	0.157^***^(5.748)	2.84
PFE	0.142^***^(4.382)	2.67
IRE	0.129^***^(4.117)	2.53
SEX	0.036^*^(1.923)	1.18
EDU	0.022^*^(1.964)	1.42
INC	0.051^**^(2.538)	1.76
GPF	0.038^*^(1.801)	1.59
Constant	2.138^***^(5.861)	–
*R* ^2^	0.423	
*F*	19.672	

*, **, *** represent *p* < 0.1, *p* < 0.05, and *p* < 0.01, respectively.

For the control variables, the results reveal that gender, education level, income status, and attention to green policies all have positive impacts on green consumption behavior. Specifically, female consumers exhibit a higher propensity for engagement in green consumption, while higher education levels and economic capacity enhance individuals’ understanding of green concepts and willingness to adopt sustainable practices. Additionally, attention to green policies is shown to exert a guiding effect, reinforcing individuals’ identification with and commitment to pro-environmental behaviors.

To examine potential multicollinearity among the explanatory variables, variance inflation factor (VIF) tests were conducted based on the baseline regression model. The results indicate that all VIF values are below the threshold of 5. Specifically, the VIF values for the three dimensions of service experience are 2.84, 2.67, and 2.53, respectively, while the VIF values for all control variables are below 2. These findings suggest that no serious multicollinearity exists among the variables. Overall, the model estimation results demonstrate satisfactory stability and reliability.

### 4.4 Dual mediation effect test

As presented in Table 6, in the first stage of the mediation pathways, all three dimensions of service experience design (SED)—environmental perception experience (EPE), process-facilitation experience (PFE), and interaction responsiveness experience (IRE)—exerted significant positive effects on environmental awareness (EA) and emotional connection (EM). Specifically, EPE showed a coefficient of 0.216 (*t* = 6.034) on EA and 0.188 (*t* = 5.614) on EM, both significant at the 1% level. These results indicate that the ecological visualization features and environmental ambiance quality of service spaces not only function as external stimuli for direct consumer perception but also activate intrinsic recognition of environmental values, thereby inducing higher-level environmental consciousness. Concurrently, the psychological security and sense of belonging fostered by spatial environments provide critical support for emotional engagement. PFE and IRE, which reflect “interaction efficiency” and “interpersonal connection” in service processes, also demonstrated significant impacts on both EA and EM. This suggests that consumers develop recognition of green concepts through process optimization at a cognitive level, while responsive and professional service interactions strengthen psychological attachment at an emotional level.

**TABLE 6:** Mediation effect test results.

Variable	EA	EM	GCB	VIF (GCB)
EPE	0.216***(6.034)	0.188***(5.614)	0.092***(3.385)	2.78
PFE	0.193***(5.485)	0.201***(6.028)	0.084***(3.114)	2.64
IRE	0.174***(4.962)	0.157***(4.731)	0.076***(2.947)	2.53
EA	–	–	0.169***(5.228)	2.31
EM	–	–	0.153***(4.891)	2.24
Constant	2.086***(5.261)	1.972***(5.034)	1.947***(5.403)	–
R^2^	0.421	0.437	0.467	
F	18.726	19.387	21.803	

*p <0.1, **p < 0.05, ***p < 0.01.

In the second stage of the mediation pathways, both EA and EM exhibited significant positive effects on green consumption behavior (GCB), with regression coefficients of 0.169 (*t* = 5.228) and 0.153 (*t* = 4.891), respectively, both significant at the 1% level. These findings validate that service experiences translate into behavioral outcomes through the “psychological representation → behavioral motivation” logic. The underlying psychological mechanisms are not unidimensional but driven by dual rational and emotional factors. Specifically, the mediation effect of EA reflects consumers’ internalization of environmental responsibility and awareness of ecological issues during service interactions, which enhances their willingness to proactively engage in green consumption. The EM mediation pathway, in contrast, indirectly stimulates loyalty-driven behavioral responses through emotional bonds with service brands, environments, or personnel, playing a pivotal role in non-rational decision-making contexts. The combined effects of EA and EM reveal that the psychological underpinnings of green consumption behavior intertwine cognitive and affective dimensions, with distinct service-experience-triggered psychological mechanisms forming a composite driving force for behavioral decisions.

Specifically, when EA and EM were incorporated into the model, the direct effect coefficients of EPE, PFE, and IRE on GCB decreased but remained statistically significant, with values of 0.092 (*t* = 3.385), 0.084 (*t* = 3.114), and 0.076 (*t* = 2.947), respectively. These results indicate that the mediation effects played a substantial role in the model, aligning with the characteristics of partial mediation. That is, service experience design not only directly shapes consumers’ positive attitudes toward green behaviors but also exerts indirect impacts by reshaping their intrinsic psychological states. After incorporating the mediating variables, additional VIF diagnostics were conducted for the full model. The results indicate that all VIF values remain below 3, suggesting that the estimation results are not substantially affected by multicollinearity.

In addition, to ensure the robustness of the model estimation results, classical assumption tests were conducted for the final OLS regression model. The Shapiro–Wilk test yielded *W* = 0.984 (*p* = 0.063), indicating that the residuals do not significantly deviate from normality. Furthermore, the Breusch–Pagan test statistic was 6.214 (*p* = 0.183), suggesting that no significant heteroscedasticity is present.

### 4.5 Robustness test

To further validate the robustness of the model conclusions, this study employed the variable substitution method by replacing the original dependent variable “green consumption behavior (GCB)” with a “binary variable for green consumption propensity (GCB_Binary),” where consumers’ green behavior scores were categorized into high-frequency propensity (scores above 4 coded as 1) and low-frequency propensity (scores ≤ 4 coded as 0). This substitution variable more effectively differentiates the actual manifestation of green consumption behaviors, mitigates potential subjective biases in respondents’ scale ratings, and strengthens the model’s explanatory power for real-world behavioral tendencies. As shown in Table 7, even with the substituted variable, the three dimensions of service experience design—environmental perception experience (EPE), process-facilitation experience (PFE), and interaction responsiveness experience (IRE)—exhibited highly consistent directional effects and significance levels on the pathways of environmental awareness (EA) and emotional connection (EM). This confirms that the green ambiance of service settings, process convenience, and personalized interactions remain crucial drivers for activating consumers’ environmental consciousness and emotional attachment. Moreover, in the GCB_Binary model, all three service experience dimensions continued to demonstrate significant positive effects on green consumption propensity, with coefficients closely aligned with the baseline model results and stable significance levels. The mediating variables EA and EM remained significantly and positively associated with green consumption behavior, further confirming that the pathways through which service experiences indirectly influence green consumption via psychological mechanisms remain valid after variable substitution, reflecting the robustness of the mediation effects. The goodness-of-fit metrics (R^2^) and F-statistics of the overall model remained high post-substitution, indicating no substantial degradation in explanatory power after variable redefinition. These results verify the insensitivity of the path model to variable operationalization, thereby enhancing the credibility and generalizability of the research conclusions. To further ensure the estimation stability of the robustness model, VIF diagnostics were conducted for the GCB_Binary specification. The results indicate that all VIF values remain below 3, suggesting that no significant multicollinearity arises after variable substitution. This finding further supports the robustness and reliability of the study’s conclusions.

**TABLE 7:** Robustness test results.

Variable	EA	EM	GCB_Binary	VIF (GCB_Binary)
EPE	0.214^***^(5.982)	0.185^***^(5.471)	0.089^***^(3.267)	2.72
PFE	0.191^***^(5.328)	0.198^***^(5.917)	0.082^***^(3.037)	2.61
IRE	0.172^***^(4.816)	0.153^***^(4.608)	0.075^***^(2.843)	2.48
EA	–	–	0.164^***^(5.013)	2.29
EM	–	–	0.148^***^(4.732)	2.21
Constant	2.073^***^(5.194)	1.951^***^(4.993)	0.216^***^(6.034)	–
*R* ^2^	0.419	0.433	0.459	
*F*	18.263	19.014	20.186	

*p <0.1, **p < 0.05, ^***^p < 0.01.

## 5 Discussion

This study systematically explores the impact mechanisms of service experience design on green consumption behavior (GCB) by combining stepwise regression analysis with a multiple mediation effect model. Specifically, based on survey data, service experience design was decomposed into three dimensions—environmental perception experience (EPE), process-facilitation experience (PFE), and interaction responsiveness experience (IRE)—while introducing two psychological variables, environmental awareness (EA) and emotional connection (EM), to construct mediation pathways. The stepwise regression approach sequentially tested the direct effects of service experience on GCB, the explanatory effects of mediating variables, and the final dual mediation pathways. Empirical results revealed that service experience design exerts a significant positive influence on GCB, with both EA and EM serving as partial mediators, forming a dual-path mechanism driven by parallel cognitive and affective processes. These findings enrich the theoretical framework explaining green consumption behavior and provide empirical evidence for optimizing experiences in green service contexts.

This study aligns closely with existing research on green consumption behavior in multiple aspects, establishing a robust theoretical and methodological foundation. However, it further extends prior work by refining the multidimensional structure of service experience and constructing dual psychological mechanisms. Methodologically, Chang et al. (2024) employed structural equation modeling to identify the direct influence of service quality on eco-behavioral intentions in green hotel contexts, introducing perceived value as a mediator. Their study validated the causal relationship between service experience and green behavior, supporting the methodological rationale for this paper’s pathways. Building on this, the current research refines the broad concept of “perceived value” into two distinct psychological mechanisms—EA and EM—which not only better align with the intrinsic drivers of green behavior but also resonate with psychological theories on dual-system behavioral motivation, thereby enhancing the model’s theoretical completeness and explanatory precision. In terms of content design, Yu et al. (2024) decomposed green service experiences into multiple perceptual dimensions to examine their effects on green brand trust, highlighting the role of service process perceptions in shaping green behavior. This study advances that approach by categorizing service experiences into three specific dimensions and conducting quantitative analyses of their pathway relationships with dual psychological mechanisms. This treatment not only inherits the dimensional analysis tradition of service experience research but also introduces finer distinctions in measurement structures, clarifying the differentiated roles of distinct experiential elements in green consumption psychology and thereby improving the interpretability of internal structural effects within service experiences. At the conceptual level, Lumivalo et al. (2022) demonstrated that green retail environments positively stimulate consumers’ green behavioral intentions, emphasizing service experiences’ contextual priming function. The present study further argues that green service experiences foster more enduring intrinsic motivations by dual activation of cognitive identification (EA) and emotional attachment (EM). By validating the priming effects of service experiences and innovatively revealing the psychological construction processes underlying green behavior through dual mediation mechanisms, this research extends the theoretical boundaries of existing work on green service impact mechanisms.

The contributions of this study lie in advancing green consumption research from a product- or policy-centric paradigm to a service-process-centric paradigm, broadening the understanding of “behavioral incentive mechanisms” in sustainable consumption. By integrating theoretical insights from management science, behavioral science, and environmental psychology, this study constructs a theoretical framework linking service experiences to green behavior, thereby strengthening the theoretical foundation and interdisciplinary value of green service research. The findings provide actionable strategic guidance for optimizing green services and implementing behavior-guided management practices, offering practical implications for corporate green transitions, green business model design, and innovation in public service delivery mechanisms.

However, this study has limitations. First, its reliance on cross-sectional survey data limits the ability to capture dynamic variations or time-lagged effects in variable relationships. Second, the sample was primarily drawn from consumers in green consumption contexts, who may inherently exhibit relatively higher levels of environmental awareness and pro-environmental attitudes. This may introduce a degree of sample selection bias. Therefore, caution should be exercised when generalizing the findings to the broader consumer population; furthermore, data collection depended on self-reported measures, which might be influenced by social desirability bias or cognitive distortions, introducing subjectivity. Finally, while the model systematically incorporates multiple variable dimensions, its control over complex factors such as individual heterogeneity and social network effects remains limited, potentially affecting explanatory validity.

## 6 Conclusion

This study leverages questionnaire data and employs regression alongside mediation modeling to systematically examine the cognitive and affective processes linking service experiences to sustainable consumption. Core insights reveal:

According to the study, service experience design plays a crucial role in encouraging green purchasing decisions. A service environment’s eco-friendly atmosphere, streamlined procedures, and prompt staff engagement all contribute significantly to boosting consumers’ sustainable choices. The regression analysis yields coefficients of 0.157 (*t* = 5.748) for environmental perception, 0.142 (*t* = 4.382) for procedural efficiency, and 0.129 (*t* = 4.117) for interaction quality. This evidence highlights how effective service design can inherently steer consumers toward greener habits, reinforcing the theoretical link between service quality and sustainable consumption transitions.

The study further uncovers that environmental concern and emotional resonance serve as key mediators between service experience and green behavior, creating a two-path system fueled by thought and feeling. Each aspect of service experience substantially boosts both mediators at *p* < 0.01. For instance, environmental perception registers effects of 0.216 (*t* = 6.034) and 0.188 (*t* = 5.614), whereas the mediators’ impact on green consumption is 0.169 (*t* = 5.228) and 0.153 (*t* = 4.891). Even after accounting for mediation, service experience retains a significant (though reduced) direct effect, confirming partial mediation. Thus, service experiences drive sustainable behavior both directly and indirectly by heightening ecological awareness and personal connection.

The study uncovers the intrinsic mechanism through which service experience design influences green consumption behavior via parallel cognitive and affective pathways. Future research may further deepen and extend this line of inquiry in several respects. At the data-structure level, constructing longitudinal tracking databases or employing scenario-based experimental designs would enable continuous observation of the dynamic relationship between service experience and green consumption behavior, thereby strengthening causal inference from a temporal perspective.

In terms of sample selection, future studies could broaden the respondent base to include individuals with varying levels of environmental awareness and diverse consumption backgrounds. By adopting stratified sampling strategies or cross-regional comparative designs, researchers may examine the stability of the proposed model across a wider population, thus enhancing the external validity of the findings. Additionally, integrating multiple data collection approaches—such as behavioral tracking, physiological measurements, or scenario simulations—would improve data objectivity and reliability while reducing self-report bias. Furthermore, incorporating external variables such as individual heterogeneity and social network structures into the analytical framework would allow for a more comprehensive understanding of how service experience shapes green behavior across different demographic groups and social contexts, thereby strengthening both the explanatory depth and generalizability of the research conclusions.

## Data Availability

The original contributions presented in this study are included in the article/supplementary material, further inquiries can be directed to the corresponding author.
